# Indoleamine 2,3-dioxygenase-expressing dendritic cells are involved in the generation of CD4^+^CD25^+ ^regulatory T cells in Peyer's patches in an orally tolerized, collagen-induced arthritis mouse model

**DOI:** 10.1186/ar2361

**Published:** 2008-01-25

**Authors:** Min-Jung Park, So-Youn Min, Kyung-Su Park, Young-Gyu Cho, Mi-La Cho, Young-Ok Jung, Hyun-Sil Park, Soog-Hee Chang, Seok Goo Cho, Jun-Ki Min, Sung-Hwan Park, Ho-Youn Kim

**Affiliations:** 1 The Rheumatism Research Center, Catholic Research Institute of Medical Science, The Catholic University of Korea, Banpo-dong, Seocho-gu, Seoul 137-701, South Korea; 2 Center for Rheumatic Disease, Division of Rheumatology, Department of Internal Medicine, Kangnam St Mary's Hospital, The Catholic University of Korea, Banpo-dong, Seocho-gu, Seoul 137-701, South Korea; 3 Department of Hematology, Catholic Hematopoietic Stem Cell Transplantation, Youido St Mary's Hospital. The Catholic University of Korea, Youido-dong, Youngdungpo-Gu, Seoul 150-713, South Korea

## Abstract

**Introduction:**

The present study was devised to understand the role of systemic indoleamine 2,3-dioxygenase (IDO) in the tolerance induction for orally tolerized mice in collagen-induced arthritis (CIA). We examined whether IDO-expressing dendritic cells (DCs) are involved in the generation of CD4^+^CD25^+ ^regulatory T cells during the induction of oral tolerance in a murine CIA model.

**Methods:**

Type II collagen was fed six times to DBA/1 mice beginning 2 weeks before immunization, and the effect on arthritis was assessed. To examine the IDO expression, the DCs of messenger RNA and protein were analyzed by RT-PCR and Flow cytometry. In addition, a proliferative response assay was also carried out to determine the suppressive effects of DCs through IDO. The ability of DCs expressing IDO to induce CD4^+^CD25^+ ^T regulatory cells was examined.

**Results:**

CD11c^+ ^DCs in Peyer's patches from orally tolerized mice expressed a higher level of IDO than DCs from nontolerized CIA mice. IDO-expressing CD11c^+ ^DCs were involved in the suppression of type II collagen-specific T-cell proliferation and in the downregulation of proinflammatory T helper 1 cytokine production. The suppressive effect of IDO-expressing CD11c^+ ^DCs was mediated by Foxp3^+^CD4^+^CD25^+ ^regulatory T cells.

**Conclusion:**

Our data suggest that tolerogenic CD11c^+ ^DCs are closely linked with the induction of oral tolerance through an IDO-dependent mechanism and that this pathway may provide a new therapeutic modality to treat autoimmune arthritis.

## Introduction

Repeated oral administration of autoantigen can suppress autoimmune responses in collagen-induced arthritis (CIA) and experimental autoimmune encephalomyelitis, and can suppress diabetes in nonobese diabetic mice [1-10]. Although the mechanisms responsible for the induction of oral tolerance have not been elucidated fully, repeated oral administration of a high dose of antigen can induce oral tolerance by anergy or deletion of antigen-specific T cells. In contrast, repeated feeding of a low dose of antigen favors the induction of active immune regulation involving regulatory T cells, including transforming growth factor beta (TGFβ)-producing T helper 3 cells, IL-10-producing T regulatory 1 cells, and CD4^+^CD25^+ ^T cells [1,10,11]. Previous studies have demonstrated that, after repeated oral administration of type II collagen (CII) and subsequent induction of CIA, the mean arthritis index is lower in tolerized mice than in CIA mice [12] and the proportion of IL-10-producing CD4^+^CD25^+ ^T cells increases in Peyer's patches and spleens of tolerized mice [13]. Among the various immune cells involved in the induction of oral tolerance, dendritic cells (DCs) may play a major role in linking orally administered antigen to antigen-specific tolerance.

DCs are professional antigen-presenting cells (APCs) that play a decisive role in determining immunity or immune tolerance; this determination is based on the maturation or activation state and the subset of DCs, and cytokine profiles in the microenvironment at the time of antigen uptake [1,14-16]. A previous study demonstrated that CD11c^+^CD11b^+ ^DCs, which increase in number in Peyer's patches during the induction of tolerance to CII, suppress T-cell proliferation and induce CD4^+^CD25^+ ^regulatory T cells. CD11c^+^CD8α^+ ^DCs, however, promote T-cell proliferation [12]. The mechanisms underlying the suppression by DCs of the expansion and differentiation of effector T cells and promotion of T-cell tolerance remain elusive.

One regulatory mechanism of DCs is the suppression of proliferation by producing the enzyme indoleamine 2,3-dioxygenase (IDO), which degrades the essential amino acid tryptophan. Murine macrophages and DCs expressing IDO inhibit T-cell proliferation or induce T-cell apoptosis *in vitro *and *in vivo *[17]. Munn and colleagues reported that plasmacytoid DCs in tumor-draining lymph nodes express IDO constitutively, which causes local immunosuppression and T-cell anergy *in vivo *[18,19]. Mellor and colleagues reported that systemic administration of CpG oligodeoxynucleotides induces IDO expression in splenic CD19^+ ^DCs, which acquire regulatory functions in an IDO-dependent manner [20]. Upon exposure to allergen within the mucosa, Langerhans-like DCs expressing high-affinity IgE receptors produce IL-10 and TGFβ, upregulate IDO expression, and suppress the allergic response in humans [16,21,22]. Whether the tolerogenic activity of DCs from Peyer's patches in orally tolerized mice is IDO dependent, however, is unknown.

To elucidate the expression of IDO and its role in the induction of oral tolerance, we prepared DCs from Peyer's patches of DBA/1 mice after induction of oral tolerance by repeated oral administration of CII and subsequent induction of CIA. We examined whether IDO-expressing DCs have tolerogenic characteristics and whether they can induce CD4^+^CD25^+ ^regulatory T cells. Our results demonstrate that IDO-expressing DCs in Peyer's patches play an essential role in the induction of oral tolerance in this model of autoimmune disease.

## Materials and methods

### Animals

Six-week-old to 8-week-old male DBA/1J mice (SLC, Inc., Shizuoka, Japan) were maintained in groups of two to four animals in polycarbonate cages in a specifically pathogen-free environment and were fed standard mouse chow (Ralston Purina, St Louis, MO, USA) and water *ad libitum*. All experimental procedures were examined and approved by the Animal Research Ethics Committee at the Catholic University of Korea.

### Preparation of type II collagen

Bovine CII was kindly provided by Professor Andrew Kang of the University of Tennessee. CII was extracted in its native form from the fetal calf articular cartilage and was purified as described previously [23].

### Induction of oral tolerance in DBA/1 mice

DBA/1 mice were sacrificed either with 100 μg bovine CII dissolved in 0.05 N acetic acid and 4 mg/ml solution of 25 μl CII solution + 175 μl PBS for the tolerance group or with an equal volume of an acetic acid–PBS mixture (25 μl of 0.05 N acetic acid + 175 μl PBS) for the CIA group. Administration was performed using an oral Zonde needle (Natsume, Tokyo, Japan) every 2 days over 2 weeks, beginning 2 weeks before immunization.

### Induction and evaluation of arthritis

Bovine CII was dissolved in 0.05 N acetic acid to 4 mg/ml concentration and was emulsified (1:1 ratio) with complete Freund's adjuvant. As a primary immunization, 0.1 ml emulsion containing 100 μg CII was injected into the tail. Two weeks later, a booster injection of 100 μg CII dissolved similarly and emulsified 1:1 with incomplete Freund's adjuvant was administered to the hind leg.

Starting 18 days after the primary immunization, three independent observers examined the severity of arthritis three times a week for up to 11 weeks. The severity of arthritis was recorded as the mean arthritic index on a 0 to 4 scale according to the following criteria [24]: 0 = no edema or swelling, 1 = slight edema and erythema limited to the foot or ankle, 2 = slight edema and erythema from the ankle to the tarsal bone, 3 = moderate edema and erythema from the ankle to the tarsal bone, and 4 = edema and erythema from the ankle to the entire leg. The sum of the values from three legs, excluding the hind leg into which CII–incomplete Freund's adjuvant was injected, was determined and divided by three to obtain an average. The final value represents the average recorded by three independent observers.

### Isolation of dendritic cells

Mononuclear cells from Peyer's patches were incubated with anti-mouse CD11c-coated magnetic beads (Miltenyi Biotec, Auburn, CA, USA) and then subjected to positive selection through magnetic-activated cell sorting. Separated cells routinely showed >98% viable DCs.

### Determination of the type II collagen-specific T-cell proliferative response

Mice were killed 5 weeks after primary immunization. The Peyer's patches were removed, treated for 90 minutes at 37°C with media containing dithiothreitol and ethylenediamine tetraacetic acid to remove epithelial cells, and washed extensively with Hanks' balanced salt solution. The Peyer's patches were then digested with collagenase D and DNase, and were incubated in the presence of 5 mM ethylenediamine tetraacetic acid for 5 minutes at 37°C. Prepared mononuclear cells were then plated in 96-well microtiter plates at a concentration of 2 × 10^5 ^cells/well and cultured for 3 days with 40 μg/well CII in 0.3 ml Click's medium supplemented with 0.5% mouse serum. CD11c^+ ^DCs (1 × 10^4 ^cells) isolated from Peyer's patch mononuclear cells of tolerized or CIA mice were cultured for 3 days with CII-reactive CD4^+ ^T cells (1 c 10^5 ^cells) and irradiated APCs (1 × 10^5 ^cells) obtained from Peyer's patch cells of CIA mice. Cells were pretreated with the IDO-specific inhibitor 1-methyl tryptophan (1-MT) (200 μM) for 2 hours before CII stimulation. Eighteen hours before the termination of culture, 0.5 μCi [^3^H]thymidine (New England Nuclear, Boston, MA, USA) was added to each well. Cells were harvested onto glass fiber filters and were counted in a Matrix-96 direct ionization counter (Packard Instrument Co., Downers Grove, IL, USA). Data are presented as the mean counts per minute (cpm) of triplicate cultures.

Various numbers of CD4^+^CD25^+ ^T cells that had been expanded by exposure to CD11c^+ ^DCs from Peyer's patches of tolerized mice in the presence of CII stimulation were cultured for 3 days in the presence of CII (40 μg/well) with CII-reactive CD4^+ ^T cells (1 × 10^5 ^cells) and irradiated APCs (1 × 10^5 ^cells) obtained from CIA mice. Proliferative responses were measured as the amount of [^3^H]thymidine incorporated during the last 18 hours of incubation.

### Reverse transcription–polymerase chain reaction analysis of indoleamine 2,3-dioxygenase and Foxp3 expression

Total RNA (2 μg) was reverse transcribed into cDNA using a transcription kit (TaqMan Reverse Transcription Reagents; Applied Biosystems, Darmstadt, Germany). The resulting cDNA was amplified by PCR using IDO sense (5'-CACTGTACCAGTGCAGTAG-3') and antisense (5'-ACCATTCACACACT CGTTAT-3') primers, and using Foxp3 sense (5'-CAGCTGCCTACAGTGCCCCTAG-3') and antisense (5'-CATTTGCCACGAGTGGGTAG-3') primers. PCR products were separated on a 1.5% agarose gel and stained with ethidium bromide. Fragments of 472 bp for IDO and 390 bp for Foxp3 were obtained.

### Detection of cytokine production by enzyme-linked immunosorbent assay

CD4^+ ^T cells isolated from Peyer's patches of CIA mice were cocultured with CD11c^+ ^DCs from tolerized mice or CIA mice in the absence or presence of CII (40 μg/well). Cells were pretreated for 2 hours with 1-MT (200 μM). After 2 days, the culture medium was harvested from each well and stored at -70°C. The concentrations of IL-17, IL-10, and TGFβ in the culture supernatant were measured by sandwich ELISA.

### Flow cytometric analysis of intracellular indoleamine 2,3-dioxygenase and regulatory T cells

Single mononuclear cells were prepared from Peyer's patches of tolerized and CIA mice, stained with Fluorescein isothiocyanate-labeled anti-CD11c mAb, permeabilized, and fixed with CytoPerm/CytoFix (Pharmingen, BD, San Diego, CA, USA) as instructed by the manufacturer. Cells were stained further with rabbit anti-IDO polyclonal antibody (Transgenic Inc, Kobe, Japan), followed by Phycoerythrin-conjugated goat anti-rabbit immunoglobulin, and then subjected to flow cytometric analysis (FACSCalibur; Becton Dickinson, San Jose, CA, USA). Rabbit IgG was used as the corresponding isotype antibody control.

To isolate CD4^+^CD25^- ^T cells, mononuclear cells from Peyer's patches from tolerized mice were stained with a mixture of anti-CD4 PerCP and CD25 allophycocyanin mAbs (Pharmingen, BD) and were sorted using the Vantage FACSorter (BD Bioscience, San Diego, CA). The purity of the sorted cells was 95% to 99% as evaluated by flow cytometry. CD11c^+ ^DCs (1 × 10^4 ^cells) isolated from Peyer's patch mononuclear cells of tolerized or CIA mice were cultured for 3 days with CD4^+^CD25^- ^T cells (1 × 10^5 ^cells) obtained from Peyer's patch cells of tolerized mice in the presence or absence of CII. To measure the amount of intracellular Foxp3 in CD4^+^CD25^+ ^T cells, CD4^+ ^T cells and DCs were cocultured and surface-stained with PerCP-labeled anti-mouse CD4 and allophycocyanin-labeled anti-mouse CD25, and then with 0.5 μg Phycoerythrin-conjugated anti-mouse Foxp3 or Phycoerythrin-conjugated rat IgG2a isotype control using the regulatory T Cell Staining Kit (eBioscience, San Diego, CA, USA).

### Confocal microscopy

Intracellular immunofluorescence staining was performed using the intracellular flow cytometric method described above. The cells were allowed to adhere to the glass slide using a Shandon CytoSpin III cytocentrifuge (GMI, Ramsey, MN, USA). The slides were then mounted using fluorescent mounting medium (Dako, Trappes, France). Confocal analysis was performed with a confocal laser scanning microscope (LSM 510 meta; Carl Zeiss, Heidelberg, Germany) equipped with a krypton–argon mixed-gas laser as the light source.

### Statistical analysis

The arthritis scores at the different times were compared between the two groups using the nonparametric Mann–Whitney *U *test. All data are expressed as the mean ± standard deviation. Statistical analysis was performed using SPSS 10.0 for Windows (SPSS, Chicago, IL, USA). The differences between groups were analyzed using an unpaired Student's *t *test, assuming equal variances. *P *< 0.05 was considered significant.

## Results

### Repeated oral administration of type II collagen induces immune tolerance and inhibits arthritis development

**Figure 1:**
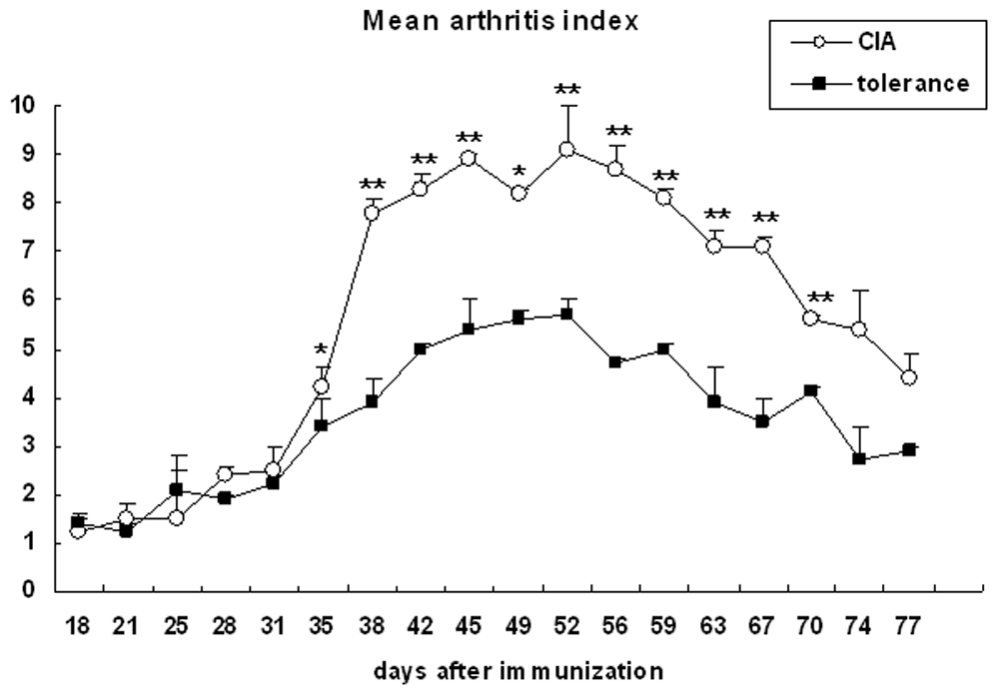
Inhibition of arthritis development in tolerized mice. Mice in the tolerance group were fed 100 μg type II collagen (CII) six times for 2 weeks before immunization. For collagen-induced arthritis (CIA) induction, CII emulsified with complete Freund's adjuvant was injected into the tail of mice in the tolerance group and in the CIA group as a primary immunization. Two weeks later, CII emulsified with incomplete Freund's adjuvant was injected into a hind leg as a booster injection. The mean arthritis index was significantly lower in the tolerance group than in the CIA group throughout the examination period. Values are presented as the mean ± standard deviation of three independent experiments involving 20 tolerized mice and 20 CIA mice per group. *P < 0.05, **P < 0.005.

The arthritis index remained low in both the tolerance and CIA groups until 4 weeks after primary immunization with CII–complete Freund's adjuvant. In the CIA group, the arthritis index began to increase after week 5, reached a peak between weeks 6 and 9 after primary immunization, and then decreased by week 11. In the tolerance group, the arthritis index peaked between weeks 6 and 9, but the index was significantly lower than that of the CIA group throughout the examination period (Figure 1).

### Induction of oral tolerance increases the proportion of indoleamine 2,3-dioxygenase-expressing CD11c^+ ^dendritic cells in Peyer's patches of tolerized mice

DCs are potent stimulators of naïve T cells and are key inducers of immune tolerance [25]. One molecular mechanism by which DCs regulate T cells is through the expression of IDO, which degrades the essential amino acid tryptophan [26]. To determine whether IDO expression increases in tolerized mice, we examined the relative proportion of IDO^+ ^DCs among CD11c^+ ^DCs in Peyer's patches after repeated oral administration of CII and subsequent CIA induction.

**Figure 2:**
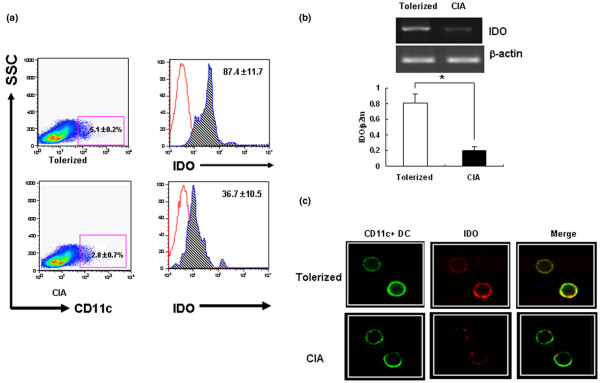
Oral tolerance induction in indoleamine 2,3-dioxygenase-expressing CD11c^+ ^dendritic cells of tolerized mice. The induction of oral tolerance increases the proportion of indoleamine 2,3-dioxygenase (IDO)-expressing CD11c^+ ^dendritic cells (DCs) in Peyer's patches of tolerized mice. **(a) **Flow cytometric analysis of IDO in CD11c^+ ^DCs isolated from Peyer's patches. Mononuclear cells obtained from Peyer's patches of tolerized mice and of CIA mice were probed with Fluorescein isothiocyanate-labeled anti-CD11c mAb and were fixed with CytoPerm/CytoFix for 20 minutes. Cells were probed for intracellular IDO using anti-mouse IDO antibody and were analyzed by flow cytometry. The histograms were gated on CD11c^+ ^DCs. Dotted histogram lines represent cells stained with isotype-matched control monoclonal antibodies. Results are the mean ± standard deviation of replicate samples from seven independent experiments. SSC. **(b) **Analysis of IDO transcription in tolerized mice and CIA mice. CD11c^+ ^DCs were isolated from Peyer's patch mononuclear cells using the magnetic-activated cell sorting system. The expression of IDO mRNA was analyzed using RT-PCR. β_2_-Actin was used as an internal control. Each value is the mean ± standard deviation of replicate determinations in one of four experiments. *P < 0.05. **(c) **Immunofluorescent confocal microscopic examination of IDO expression by CD11c^+ ^DCs. Mononuclear cells obtained from Peyer's patches of tolerized and CIA mice were stained with Fluorescein isothiocyanate-labeled anti-CD11c (green) and anti-IDO (red), fixed, and were examined using confocal microscopy. Isotype-matched control antibody staining was negative (data not shown). Data are representative of three independent experiments.

IDO expression was significantly higher in DCs from tolerized mice than in CIA mice (mean fluorescence index, 87.4 ± 11.2 versus 36.7 ± 10.2, *P *< 0.05). Normal DBA/1 mice had the lowest mean fluorescence index of IDO^+ ^DCs (10.2 ± 5.2, *P *< 0.05; data not shown) (Figure 2a). These data were confirmed by measuring the mRNA level of IDO in CD11c^+ ^DCs obtained from Peyer's patches of tolerized and CIA mice. We used semiquantitative RT-PCR to measure IDO mRNA expression in CD11c^+ ^DCs isolated from Peyer's patches. The IDO transcripts were upregulated markedly in tolerized mice compared with CIA mice (Figure 2b).

Using confocal microscopy, we identified the expression of IDO *in situ *at the single-cell level. Single cells were stained simultaneously with a pandendritic cell marker, anti-CD11c (green), and an IDO-specific antibody (red). About one-third of the CD11c^+ ^DCs from tolerized mice expressed IDO (yellow). In contrast, the DCs from CIA mice rarely expressed IDO (Figure 2c).

### Indoleamine 2,3-dioxygenase-expressing CD11c^+ ^dendritic cells have an immature phenotype

Human IDO^+ ^DCs show a mature phenotype characterized by CD14^-^, CD83^+^, CD80^+^, CD86^high^, and HLA-DR^high ^[27,28]. In contrast, DCs expressing IDO in mice show an immature phenotype and suppress T-cell proliferation both *in vitro *and *in vivo*. The maturational status of IDO-expressing DCs in Peyer's patches of tolerized mice, however, has never been considered.

**Figure 3:**
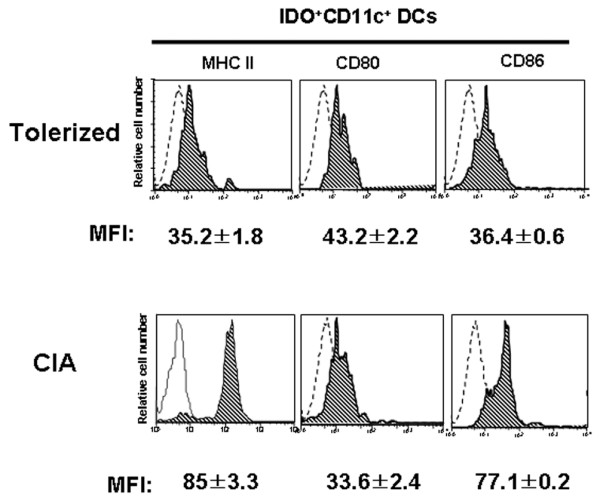
Indoleamine 2,3-dioxygenase-expressing CD11c^+ ^dendritic cells display a phenotype consistent with the immature state. Indoleamine 2,3-dioxygenase (IDO)^+^CD11c^+ ^dendritic cells (DCs) were stained for markers of DC maturity and were analyzed using three-color flow cytometry. Mononuclear cells from Peyer's patches were stained for CD11c, IDO, and Major Histocompatibility Complex (MHC) II, or costimulatory molecule markers. All plots were first gated on IDO^+^CD11c^+ ^cells. Dotted line shows the isotype-matched controls. Data are the mean ± standard deviation of three experiments. MFI, mean fluorescence index.

We examined the expression of major histocompatibility complex II, CD80, and CD86 by CD11c^+ ^IDO^+ ^DCs from tolerized mice and CIA mice. CD11c^+ ^IDO^+ ^DCs from tolerized mice expressed low levels of MHC II and CD86, suggesting an immature phenotype. In contrast, the expression of these surface molecules was significantly higher on DCs from CIA mice, which is characteristic of mature DCs (Figure 3). The expression of CD80, however, did not differ significantly between the two groups.

### CD11c^+ ^dendritic cells of tolerized mice inhibit type II collagen-specific T-cell proliferation in an indoleamine 2,3-dioxygenase-dependent manner

APC-induced T-cell activation requires contact-dependent bidirectional signaling between the APCs and the T cells [29]. This signaling may upregulate IDO expression in DCs, and this may control autoreactive T cells by depleting tryptophan [25,30]. We explored whether IDO affects the ability of DCs isolated from Peyer's patches of tolerized mice to regulate T-cell responses. Mixed lymphocyte cultures were performed in the presence or absence of the IDO-specific inhibitor 1-MT. CD11c^+ ^DCs from Peyer's patches of tolerized mice or CIA mice were cocultured for 3 days with CII-reactive CD4^+ ^T cells and irradiated APCs obtained from CIA mice in the absence or presence of CII.

**Figure 4:**
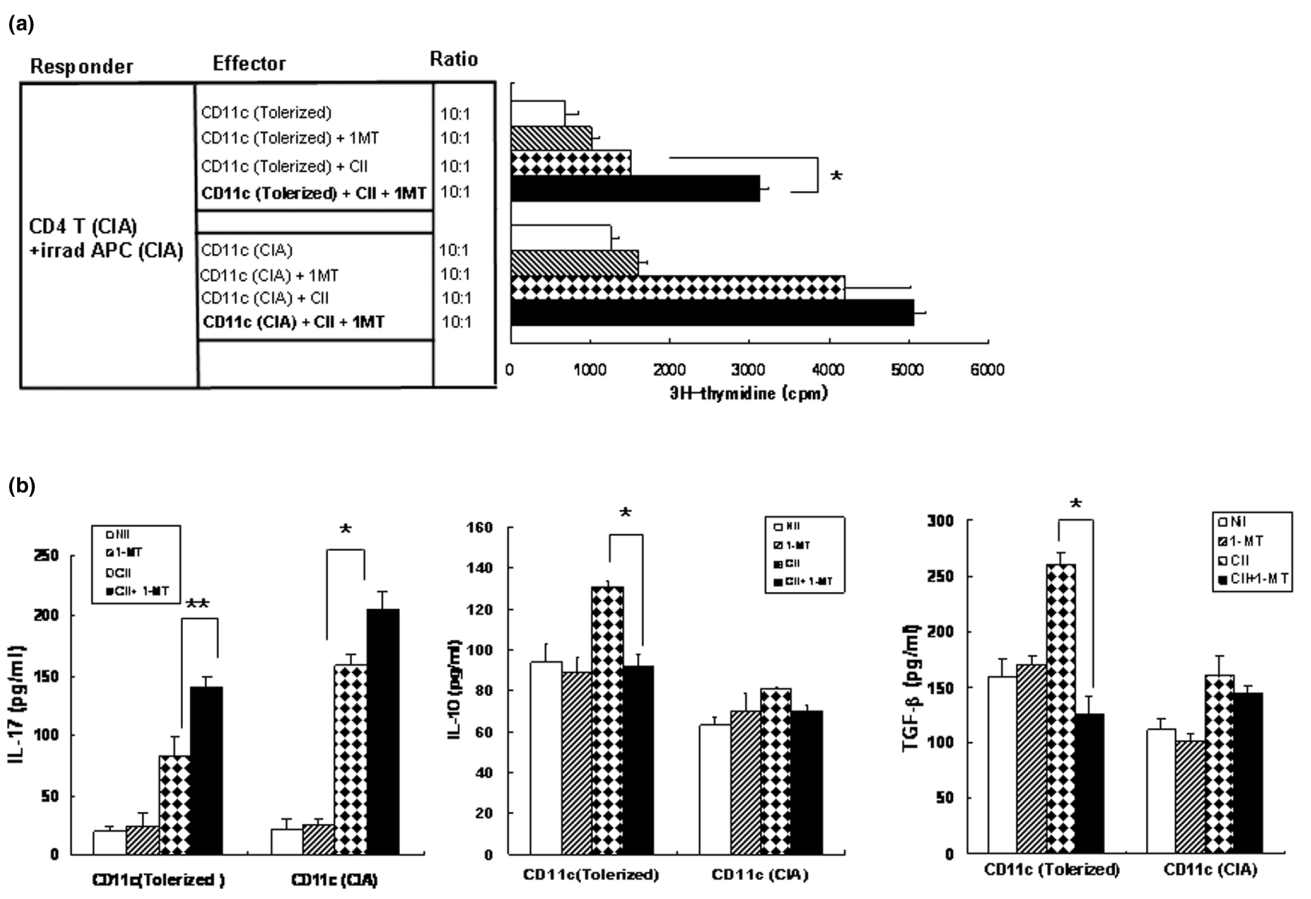
Tolerized mice CD11c^+ ^dendritic cells induce indoleamine 2,3-dioxygenase-dependent inhibition of type II collagen-specific T-cell proliferation. **(a) **Type II collagen (CII)-reactive CD4^+ ^T cells (1 × 10^5^/well) and irradiated antigen-presenting cells (APCs) (1 × 10^5^/well) isolated from Peyer's patches of collagen-induced arthritis (CIA) mice were cultured with CD11c^+ ^dendritic cells (DCs) (1 × 10^4^/well) from tolerized mice or CIA mice in the presence or absence of CII (40 μg/ml) in 96-well, U-bottomed plates for 3 days. Some DCs were pretreated with 1-methyl tryptophan (1-MT) (200 μM) for 2 hours before coculture. Data presented as the mean counts per minute (cpm) of triplicate cultures. Values are the mean ± standard deviation from three independent experiments. *P < 0.05. **(b) **Cytokine concentrations in the coculture supernatants. Concentrations of IL-17, IL-10, and transforming growth factor beta (TGFβ) in the culture supernatants were measured by ELISA. Values are the mean ± standard deviation from three independent experiments. *P < 0.05, **P < 0.001.

Without CII stimulation, the proliferation of CII-reactive CD4^+ ^T cells was inhibited more by CD11c^+ ^DCs from Peyer's patches of tolerized mice than by CD11c^+ ^DCs from Peyer's patches of CIA mice (687 ± 159 cpm versus 1,257 ± 103 cpm, *P *< 0.05) (Figure 4a). With CII-specific stimulation, the suppressive effect was more prominent in CD11c^+ ^DCs from tolerized mice. As shown in Figure 4a, proliferation of CD4^+ ^T cells induced by CD11c^+ ^DCs of tolerized mice was suppressed to be about one-third of that in CD4^+ ^T cells cultured with CD11c^+ ^DCs of CIA mice (1,507 ± 817 cpm versus 4,204 ± 95 cpm, *P *< 0.05). Addition of the IDO inhibitor 1-MT, however, abolished the suppressive effect of CD11c^+ ^DCs from tolerized mice on CII-specific T-cell proliferation (1,507 × 10^3 ^± 817 cpm versus 3,128 × 10^3 ^± 101 cpm, *P *< 0.05). Without CII stimulation, 1-MT had no significant effect on T-cell proliferation in either group.

To investigate the effect of IDO on the suppression of antigen-specific T cells exerted by CD11c^+ ^DCs from tolerized mice, cytokine concentrations were measured in the coculture supernatants. The IL-17 concentration was significantly lower in the culture supernatants of CD11c^+ ^DCs of tolerized mice than in the supernatants of CD11c^+ ^DCs of CIA mice cultured in the presence of CII (82 ± 17 pg/ml versus 158 ± 9 pg/ml, *P *< 0.05) (Figure 4b). Addition of 1-MT into the coculture in the presence of CII, however, markedly increased the production of IL-17 by tolerized CD11c^+ ^DCs (from 82 ± 17 pg/ml to 141 ± 15 pg/ml, *P *< 0.01). The concentrations of anti-inflammatory cytokines such as IL-10 and TGFβ were higher in the culture supernatants of CD11c^+ ^DCs of tolerized mice than in the supernatants of CD11c^+ ^DCs of CIA mice (IL-10, 131 ± 14 pg/ml versus 79 ± 13 pg/ml (*P *< 0.05); and TGFβ, 260 ± 11 pg/ml versus 195 ± 16 pg/ml (*P *< 0.05)). The addition of 1-MT, however, significantly decreased the production of IL-10 and TGFβ in the tolerance group (IL-10, from 131 ± 14 pg/ml to 92 ± 16 pg/ml (*P *< 0.05); and TGFβ, from 260 ± 11 pg/ml to 126 ± 9 pg/ml (*P *< 0.05)), suggesting that IDO expression on CD11c^+ ^DCs plays an important role in immune suppression.

### Indoleamine 2,3-dioxygenase-dependent induction of antigen-specific CD4^+^CD25^+ ^T cells by CD11c^+ ^dendritic cells in tolerized mice

Regulatory APCs may form a bridge between regulatory T cells and responder T cells, and this has been proposed as a mechanism contributing to the phenomenon of linked suppression and dominant tolerance [26]. Experimental evidence indicates that murine regulatory T cells can induce the expression of IDO [31]. We hypothesized that CD11c^+ ^DCs in tolerized mice are likely to be the IDO-dependent biologically relevant trigger for the generation or conversion of CD4^+^CD25^+ ^regulatory T cells. In a previous study, CD11c^+^CD11b^+ ^DCs isolated from Peyer's patches of tolerized mice seemed necessary for the expansion and differentiation of CD4^+^CD25^+ ^T cells, which suppress CII-specific T-cell proliferation [12]. Highly purified CD4^+^CD25^- ^T cells isolated from tolerized mice (purity > 99%) were cocultured with CD11c^+ ^DCs from tolerized or CIA mice for 3 days, without CII. The proportion of CD4^+^CD25^+ ^T cells expanded by CD11c^+ ^DCs from tolerized mice was similar to that obtained by CD11c^+ ^DCs from CIA mice (4.65% ± 0.66% versus 3.68% ± 0.46%). 1-MT had no significant effect on the proportion of CD4^+^CD25^+ ^T cells in these systems (4.74% ± 0.91% versus 3.56% ± 0.33%).

**Figure 5:**
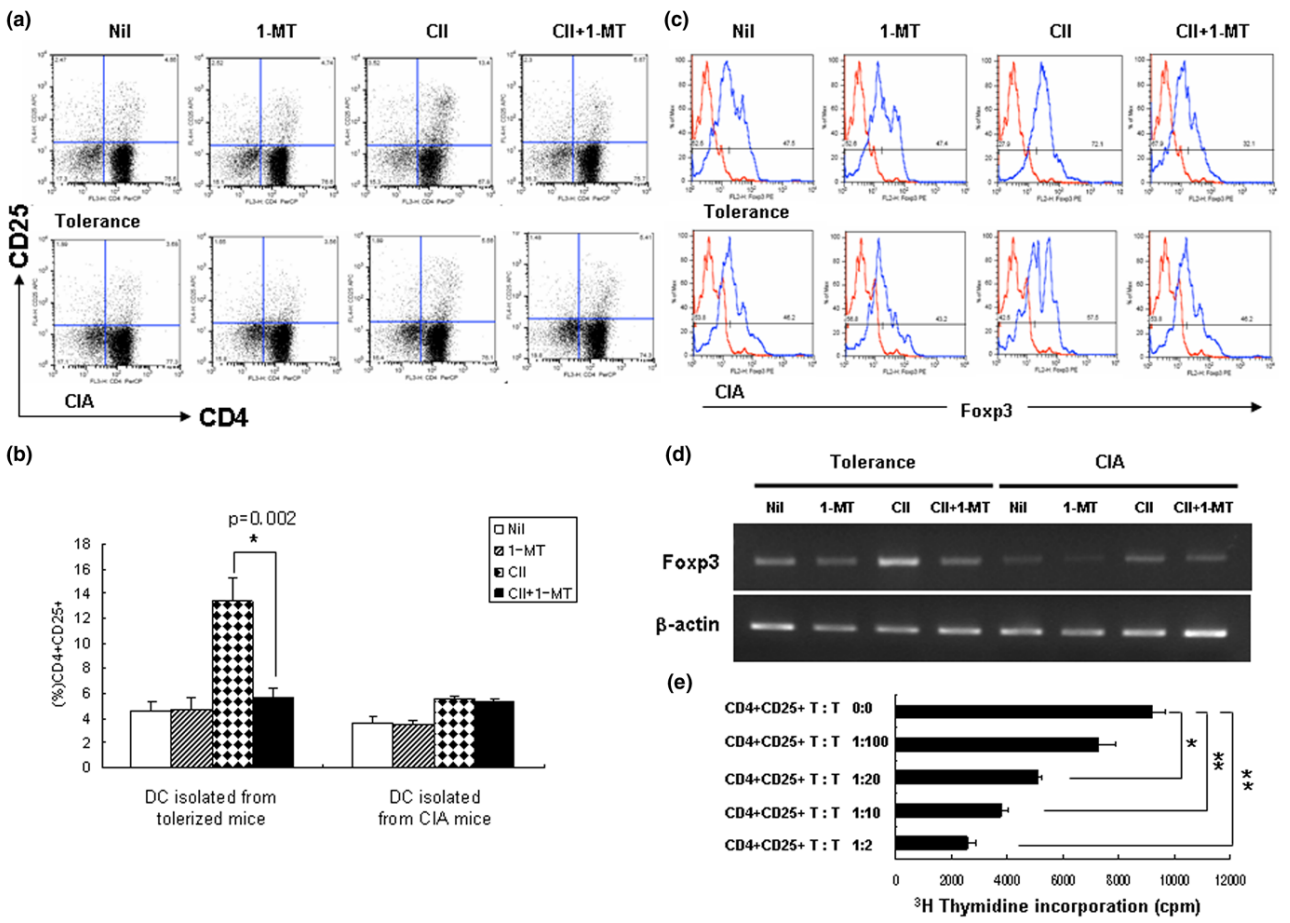
Indoleamine 2,3-dioxygenase-expressing CD11c^+ ^dendritic cells essential for antigen-specific CD4^+^CD25^+ ^regulatory T-cell induction in tolerized mice. **(a) **Increased CD4^+^CD25^+ ^T-cell proportion through an indoleamine 2,3-dioxygenase (IDO)-dependent mechanism. For regulatory T-cell induction, isolated CD4^+^CD25^- ^T cells (1 × 10^5^/well) were cultured with CD11c^+ ^dendritic cells (DCs) (2 × 10^4^/well) from tolerized mice or collagen-induced arthritis (CIA) mice in the absence or presence of type II collagen (CII) (40 μg/ml) for 3 days. 1-Methyl tryptophan (1-MT) was added to selected cultures. The proportion of CD4^+^CD25^+ ^T cells was determined using flow cytometry. Numbers represent the percentage of double-positive cells. **(b) **Summary of the percentages of CD4^+^CD25^+ ^T cells from the coculture experiments in (a). Values are the mean from four independent experiments; individual symbols are the mean in individual animals, and bars show the group means. *P < 0.02. **(c) **Analysis of Foxp3 expression by converted CD4^+^CD25^+ ^T cells. Plots were gated on CD4^+^CD25^+ ^DCs. Dotted histogram lines represent cells stained with isotype-matched control monoclonal antibodies. Data represent the mean ± standard deviation and are representative of four independent experiments. **(d) **Foxp3 mRNA expression in the same conditions as (a). β *-*Actin was used as an internal control. Results are representative of four independent experiments. **(e) **Regulatory function of the CII-induced CD4^+^CD25^+ ^T cells. CD4^+^CD25^+ ^T cells were expanded by exposure to CD11c^+ ^DCs from Peyer's patches from tolerized mice in the presence of CII antigen stimulation. Varying numbers of CD4^+^CD25^+ ^T cells were cultured for 3 days with CII-reactive CD4^+ ^T cells (1 × 10^5^) and irradiated antigen-presenting cells (1 × 10^5^) from mice with CIA in the presence of CII (40 μg/ml). Values are the mean ± standard deviation from three independent experiments. *P < 0.05, **P < 0.001. cpm, counts per minute.

We next examined whether IDO would induce naïve CD4^+^CD25^- ^T cells to differentiate into CD4^+^CD25^+ ^Foxp3^+ ^T cells in an antigen-specific manner when cocultured with CD11c^+ ^DCs. Figure 5a,b shows that the percentage of CD4^+^CD25^+ ^T cells was higher in CD4^+^CD25^- ^T cells cocultured with CD11c^+ ^DCs from tolerized mice in the presence of CII than in CD4^+^CD25^- ^T cells cocultured with CD11c^+ ^DCs from CIA mice (13.4% ± 1.89% versus 5.56% ± 0.22%, *P *< 0.05). 1-MT abrogated the increase in the proportion of CD4^+^CD25^+ ^T cells induced by CD11c^+ ^DCs from tolerized mice (5.67% ± 0.72%) but not that induced by CD11c^+ ^DCs from CIA mice (5.41% ± 0.20%). We used flow cytometry to investigate the possible conversion of CD4^+^CD25^- ^T cells into CD4^+^CD25^+ ^T cells in concomitance with Foxp3 appearance. CD4^+^CD25^+ ^T cells obtained by coculture with CD11c^+ ^DCs from tolerized mice expressed significantly higher levels of Foxp3 expression than those cells obtained by coculture with CD11c^+ ^DCs from CIA mice; this effect was diminished by the addition of 1-MT (CII stimulation, 72.1%; CII + 1-MT, 32.1%) (Figure 5c). Using RT-PCR, we also examined Foxp3 transcripts involved in the conversion of CD4^+^CD25^- ^T cells into CD4^+^CD25^+ ^T cells (Figure 5d). Our findings show that highly purified CD4^+^CD25^- ^T cells can be converted into CD4^+^CD25^+ ^Foxp3^+ ^T cells by DCs from tolerized mice by CII-specific stimulation through an IDO-dependent mechanism.

To verify that the increased population of CD4^+^CD25^+ ^Foxp3^+ ^T cells retained their suppressive function, varying numbers of CD4^+^CD25^+ ^T cells induced by exposure to CD11c^+ ^DCs from tolerized mice were cocultured with CII-reactive CD4^+ ^T cells and irradiated APCs from CIA mice for 3 days in the presence of CII. The CD4^+^CD25^+ ^T cells induced by CD11c^+ ^DCs inhibited the proliferative response of CII-reactive CD4^+ ^T cells in a concentration-dependent manner (Figure 5e).

## Discussion

Oral tolerance is initiated in the gut-associated lymphoid tissues, a well-developed immune network in the alimentary tract that comprises the mucosal epithelium, lamina propria, Peyer's patches, and mesenteric lymph nodes [1,2]. Peyer's patches are essential sites for the induction of mucosal immune responses and oral tolerance to soluble antigen, and Peyer's patch DCs are thought to play an important role in mucosal immunity and tolerance [1-4]. The selection between immunity and tolerance depends on several factors, including the occurrence of specialized DC subsets and the maturation state of the DCs [1,12]. One mechanism, exploited by tolerogenic DCs, involves IDO [17-21]. IDO-competent DCs exert regulatory effects on T cells that are mediated by tryptophan depletion and by the production of metabolic byproducts collectively known as kynurenines [16,21,22].

Demonstrating the concomitant induction of IDO enzymatic activity by DCs, our data support IDO-dependent mechanisms that have been associated with induction of T-cell tolerance and immune inhibition in the induction of oral tolerance in the murine CIA model. We examined the change in the expression of IDO in CD11c^+ ^DCs of Peyer's patches after repeated oral administration of CII and subsequent induction of CIA. In freshly isolated Peyer's patches, the proportion of CD11c^+ ^IDO^+ ^DCs was higher in tolerized mice than that in CIA mice. We found that IDO expression was induced most by oral CII feeding plus CII immunization, and that the expression of IDO did not correlate with disease severity. Next, we investigated whether the expression of IDO by DCs takes place during the initial phase of oral CII feeding or after the immunization with CII for CIA induction. In these studies, Peyer's patches were isolated from mice that had been fed with CII or PBS but not immunized with CII. We examined the expression of IDO *in situ*. The expression of IDO by DCs increased in Peyer's patches of CII-fed mice, even though these mice had not been immunized. In contrast, IDO^+ ^DCs were rare in the Peyer's patches of PBS-fed mice (data not shown). Our results show that IDO is induced by oral CII feeding and enhances the induction of immune tolerance after oral administration of antigens.

The immunosuppressive mechanism of IDO is shared by several different cell types in the immune system [30,32-36]. Splenic CD11c^+^CD19^+ ^DCs found in the mice administered CpG oligodeoxynucleotides, human monocyte-derived macrophages, and *in vitro*-derived DCs induce IDO expression and inhibit T-cell proliferation. In this regard, it will be interesting to investigate IDO expression in different subsets of DCs from tolerized mice and to characterize their role in the induction and maintenance of immune tolerance.

Tolerogenic DCs are immature, maturation-resistant, or alternatively activated DCs that express surface MHC molecules and have a low ratio of costimulatory to inhibitory signals, such as IL-10, programmed death-ligand 1, CTLA4/CD28, and IDO [37,38]. IDO expression is detected constitutively in human regulatory plasmacytoid DCs and can be induced by classical DC maturation stimuli, namely IFNγ and lipopolysaccharide or prostaglandin E_2_, which contribute to their immunoregulatory capacity [37-40]. In our study, the phenotype of DCs from tolerized mice showed an immature tolerogenic state and low levels of surface MHC II and CD86 molecules, and expressed high levels of IDO compared with DCs from CIA mice. Our study suggests that IDO is expressed constitutively in immature DCs upon repeated oral administration of CII in an animal model without artificial administration of CTLA-4 immunoglobulin or other DC-modifying agents.

Because IDO expression on DCs plays a crucial role in the induction of regulatory T cells and inhibition of the antigen-specific T-cell response, we performed mixed lymphocyte cultures to determine whether DCs isolated from Peyer's patches of tolerized mice and CIA mice have an IDO-dependent effect on CII-specific T cells. DCs from the tolerized mice inhibited the proliferation of CII-specific T cells and inflammatory cytokine production compared with DCs from CIA mice, and these suppressive effects were more evident after CII stimulation and were abrogated by addition of 1-MT, an IDO inhibitor. These findings suggest that the functional activities of IDO from tolerized mice could affect the T-cell response.

In the resting state, autoreactive T cells residing in the periphery are suppressed effectively by regulatory T cells, which are thought to prevent the development of autoimmune diseases [39]. Our group previously demonstrated that the proportion of IL-10-producing CD4^+^CD25^+ ^T cells increases more in Peyer's patches and spleens of tolerized mice than in those of CIA mice [12]. Bozza and colleagues reported that CD4^+^CD25^+ ^regulatory T cells in candidiasis are strictly dependent on the expression of B7 costimulatory molecules by IL-10-producing DCs, and are involved in the IFNγ/IDO-dependent pathway that controls the local inflammatory pathology [39]. Saito and colleagues also found that CTLA-4 on CD4^+^CD25^+ ^regulatory T cells induces the expression of IDO on DCs [40]. Considering these findings, we next sought to determine whether IDO^+ ^DCs increase the proportion of antigen-specific CD4^+^CD25^+ ^regulatory T cells in tolerized mice. Figure 5 shows that the percentage of CD4^+^CD25^+ ^cells increased significantly after coculture of CD4^+^CD25^- ^T cells with CD11c^+ ^DCs from tolerized mice in the presence of CII through an IDO-dependent mechanism. In the absence of CII, however, the proportion of CD4^+^CD25^+ ^cells cocultured with CD11c^+ ^DCs of tolerized mice was similar to that in cells cocultured with CD11c^+ ^DCs from CIA mice. This result suggests that the conversion into CD4^+^CD25^+ ^T cells without CII stimulation result from the IDO-independent expansion or selected survival of residual CD4^+^CD25^+ ^T cells. Foxp3 is a transcription factor specific for CD4^+^CD25^+ ^regulatory T cells. Foxp3 may be necessary for the induction of CD4^+^CD25^+ ^regulatory T cells by conversion of CD4^+^CD25^- ^T cells after antigen stimulation [41]. We found that CD4^+^CD25^+ ^T cells obtained after coculture of CD4^+^CD25^- ^T cells and CD11c^+ ^DCs of tolerized mice with CII expressed a large amount of Foxp3 transcript and Foxp3 protein *in vitro *in an IDO-dependent manner. In addition, the conversion of Foxp3^+^CD4^+^CD25^+ ^T cells, which are thought to be regulatory T cells, inhibited the proliferation of CII-reactive CD4^+ ^T cells in a dose-dependent manner.

CD4^+^CD25^+ ^regulatory T cells converted from peripheral CD4^+^CD25^- ^naïve T cells by TGFβ induction of Foxp3 were reported to be unresponsive to T-cell receptor stimulation, to produce neither T helper 1 nor T helper 2 cytokines, to express TGFβ, and to inhibit normal T-cell proliferation *in vitro *[42]. Different to this regulatory T-cell population, we found that the Foxp3^+^CD4^+^CD25^+ ^T cells in our model system may result partly from the expansion or selected survival of residual CD4^+^CD25^+ ^regulatory T cells and partly from the conversion of CD4^+^CD25^- ^T cells into Foxp3^+^CD4^+^CD25^+ ^regulatory T cells through an IDO-dependent mechanism.

## Conclusion

IDO expression by DCs is crucial for the induction of Foxp3^+^CD4^+^CD25^+ ^T cells and for the suppression of CII-reactive T-cell function in induction of oral tolerance to CII. These results demonstrate that the induction of IDO^+ ^DCs in Peyer's patches plays an essential role in the induction of oral tolerance and may provide a new modality of immune-based treatments for autoimmune diseases.

## Abbreviations

APC = antigen-presenting cell; bp = base pair; CIA = collagen-induced arthritis; CII = type II collagen; cpm = counts per minute; DC = dendritic cell; ELISA = enzyme-linked immunosorbent assay; IDO = indoleamine 2,3-dioxygenase; IFN = interferon; IL = interleukin; 1-MT = 1-methyl tryptophan; mAb = monoclonal antibody; MHC = major histocompatibility complex; PBS = phosphate-buffered saline; PCR = polymerase chain reaction; RT = reverse transcriptase; TGFβ = transforming growth factor beta.

## Competing interests

The authors declare that they have no competing interests.

## Authors' contributions

M-JP and S-YM performed the experimental work and prepared the manuscript. Y-GC, M-LC, K-SP, and S-GC advised on the study. H-SP, Y-OJ, and S-HC performed the experimental work H-YK is the head of the laboratory, supervised the experimental work, and advised on the study. S-HP, and J-KM are the senior researchers, and they supervised the experimental work and advised on the study. All authors read and approved the final manuscript.
